# Primary Ciliary Dyskinesia: An Update on Clinical Aspects, Genetics, Diagnosis, and Future Treatment Strategies

**DOI:** 10.3389/fped.2017.00135

**Published:** 2017-06-09

**Authors:** Virginia Mirra, Claudius Werner, Francesca Santamaria

**Affiliations:** ^1^Department of Translational Medical Sciences, Federico II University, Naples, Italy; ^2^Department of Pediatrics, Federico II University, Naples, Italy; ^3^Department of General Pediatrics, University Children’s Hospital Muenster, Muenster, Germany

**Keywords:** primary ciliary dyskinesia, Kartagener’s syndrome, bronchiectasis, ciliopathy, mucociliary clearance

## Abstract

Primary ciliary dyskinesia (PCD) is an orphan disease (MIM 244400), autosomal recessive inherited, characterized by motile ciliary dysfunction. The estimated prevalence of PCD is 1:10,000 to 1:20,000 live-born children, but true prevalence could be even higher. PCD is characterized by chronic upper and lower respiratory tract disease, infertility/ectopic pregnancy, and situs anomalies, that occur in ≈50% of PCD patients (Kartagener syndrome), and these may be associated with congenital heart abnormalities. Most patients report a daily year-round wet cough or nose congestion starting in the first year of life. Daily wet cough, associated with recurrent infections exacerbations, results in the development of chronic suppurative lung disease, with localized-to-diffuse bronchiectasis. No diagnostic test is perfect for confirming PCD. Diagnosis can be challenging and relies on a combination of clinical data, nasal nitric oxide levels plus cilia ultrastructure and function analysis. Adjunctive tests include genetic analysis and repeated tests in ciliary culture specimens. There are currently 33 known genes associated with PCD and correlations between genotype and ultrastructural defects have been increasingly demonstrated. Comprehensive genetic testing may hopefully screen young infants before symptoms occur, thus improving survival. Recent surprising advances in PCD genetic designed a novel approach called “gene editing” to restore gene function and normalize ciliary motility, opening up new avenues for treating PCD. Currently, there are no data from randomized clinical trials to support any specific treatment, thus, management strategies are usually extrapolated from cystic fibrosis. The goal of treatment is to prevent exacerbations, slowing the progression of lung disease. The therapeutic mainstay includes airway clearance maneuvers mainly with nebulized hypertonic saline and chest physiotherapy, and prompt and aggressive administration of antibiotics. Standardized care at specialized centers using a multidisciplinary approach that imposes surveillance of lung function and of airway biofilm composition likely improves patients’ outcome. Pediatricians, neonatologists, pulmonologists, and ENT surgeons should maintain high awareness of PCD and refer patients to the specialized center before sustained irreversible lung damage develops. The recent creation of a network of PCD clinical centers, focusing on improving diagnosis and treatment, will hopefully help to improve care and knowledge of PCD patients.

## Introduction

Primary ciliary dyskinesia (PCD) is a clinically and genetically heterogeneous group of disorders of ciliary motility (MIM 244400) (1). In most cases of PCD, inheritance is autosomal recessive, but X-linked PCD caused by mutations in RPGR gene, which is responsible for 20% of all cases with retinitis pigmentosa, or also in PIH1D3 gene have been reported (2–4).

History of PCD starts with Kartagener who first described a syndrome that included the triad of chronic sinusitis, bronchiectasis, and situs viscerum inversus (SI) (5). Approximately 40 years later, Afzelius reported on four subjects with recurrent bronchitis and pneumonia associated with recurrent upper airways infections who also had SI in 50% of the cases, then known as Kartagener’s syndrome (6). In that case series, sperm tails and respiratory cilia lacked dynein arms and showed impaired motility. This report clarified that a congenital defect in cilia and sperm tails can result in the association of chronic respiratory tract infections and male sterility, and the term “immotile-cilia syndrome” was eventually coined (7). The term “primary” was used to distinguish this condition from secondary ciliary abnormalities caused by inflammation and infection.

The goal of this review is to provide an update on the genetics, the diagnosis, and current and future treatment of PCD in order to increase the clinicians’ awareness of the disorder and hopefully improve final outcome.

## Cilia Biology: Structure and Function

Cilia are hair-like organelles that project from cells. Traditionally, cilia are distinguished into thre classes: primary cilia, which are not motile and are expressed on most cells during development, when they play important roles in sensing and transducing environmental signals (8); nodal cilia, which are found in the embryonic node; and motile cilia, which are long thin protrusions that extend up to 20 mm from the cell surface and propel fluids along surfaces of respiratory epithelium, brain ependyma, and falloppian tubes. Syndromes associated with defects in cilia of either classes are termed ciliopathies (9).

Each ciliated cell has approximately 200 motile cilia projecting from its surface that beat in a coordinated fashion. Motile cilia are dysfunctional in PCD. They are mainly immotile, but stiff, uncoordinated, and/or ineffective ciliary beats have also been reported (10).

Motile cilia are found in the apical surface of the upper and lower respiratory tract, on the ependymal cells that line the ventricles of the central nervous system, in the oviducts of the female reproductive system, and in the flagellum of male spermatozoa (11).

The motile cilium structure is made of nine peripheral doublet microtubules and two central single microtubules (central pair complex) and includes inner and outer dynein arms (ODAs), radial spokes, and nexin links (9 + 2 axonemes). Nexin links connect the nine peripheral doublets which are connected to the central pair by radial spokes. Outer and inner dynein arms (IDAs) are motor proteins that are attached to the outer microtubules providing energy for ciliary movement.

Cilia play a fundamental role in mucociliary clearance. Ciliary ultrastructure and orientation are critical for enhancing clearance of the lower respiratory tract as they help move fluids, mucus, and inhaled foreign materials vectorially from distal to more proximal airways. In normal airways, cilia beat with a rapid frequency that ranges from approximately 8–20 Hz and mobilizes the mucus that sits atop the cilia (12).

During embryogenesis, the motile 9 + 0 monocilia generate a whirling, rotational movement that directs leftward flow of extracellular fluid (nodal flow). The nodal cilia play a vital role in establishing left–right body orientation, and abnormalities can lead to laterality defects that include SI and a spectrum of situs ambiguous condition, that may be also associated with congenital heart abnormalities (13). The association of cilia dysfunction and SI, formerly described as Kartagener syndrome (5), may occur even in less than 50% of all PCD as some defects, in particular those associated with mutations in HYDIN, RSPH9, RSPH4A, and RSPH1 genes, do not cause SI (14).

Sperm flagella and motile cilia have a similar, although not identical, axonemal structure, which might explain why sperm flagella dyskinesia is often, but not necessarily, associated with PCD and *vice versa* (15).

## Epidemiology

In 2010, Kuehni et al. conducted the largest international survey of pediatric PCD patients ever undertaken, which included 1,192 patients from 26 European countries (16). They concluded that the prevalence of diagnosis ranged from 1:10,000 to 1:20,000 live-born children. Actually, PCD prevalence shows large variations, with estimates ranging from 1:2,200 to 1:40,000 due to different methods of analysis (17, 18). The highest prevalence was reported in Cyprus, Switzerland, and Denmark. The wide variation of doctor-diagnoses in different countries is likely due, at least in part, to geographic differences in mutational data, founder effects for certain gene mutations, high proportions of consanguineous marriages, or to differences in the diagnostic work-up of PCD among the participating countries.

Diagnosis of PCD may be delayed or missed completely, due to lack of awareness and/or difficulties in confirming it (19). In Europe, median age at diagnosis is 5.3 years, with cases with SI being confirmed as PCD at significantly lower age than those without (3.5 years versus 5.8 years) (16). Many patients may also experience a extraordinarily high number of physicians visits (50–100) before PCD is confirmed, thus indicating that also in specialized centers the awareness of the disorder may be poor (20).

Registries of patients with rare disorders are increasingly recognized as crucial tools to achieve a collection of phenotypic data, to understand the pathophysiology of the underlying condition, and to facilitate multicentre collection of data for research studies. In order to systematically collect data on PCD incidence, clinical presentation, and treatment, a registry was launched in January 2014, that provides epidemiological data and clinical information of 201 patients with PCD from several European and North-American centers (21).

## Disease Manifestations

**Figure 1:**
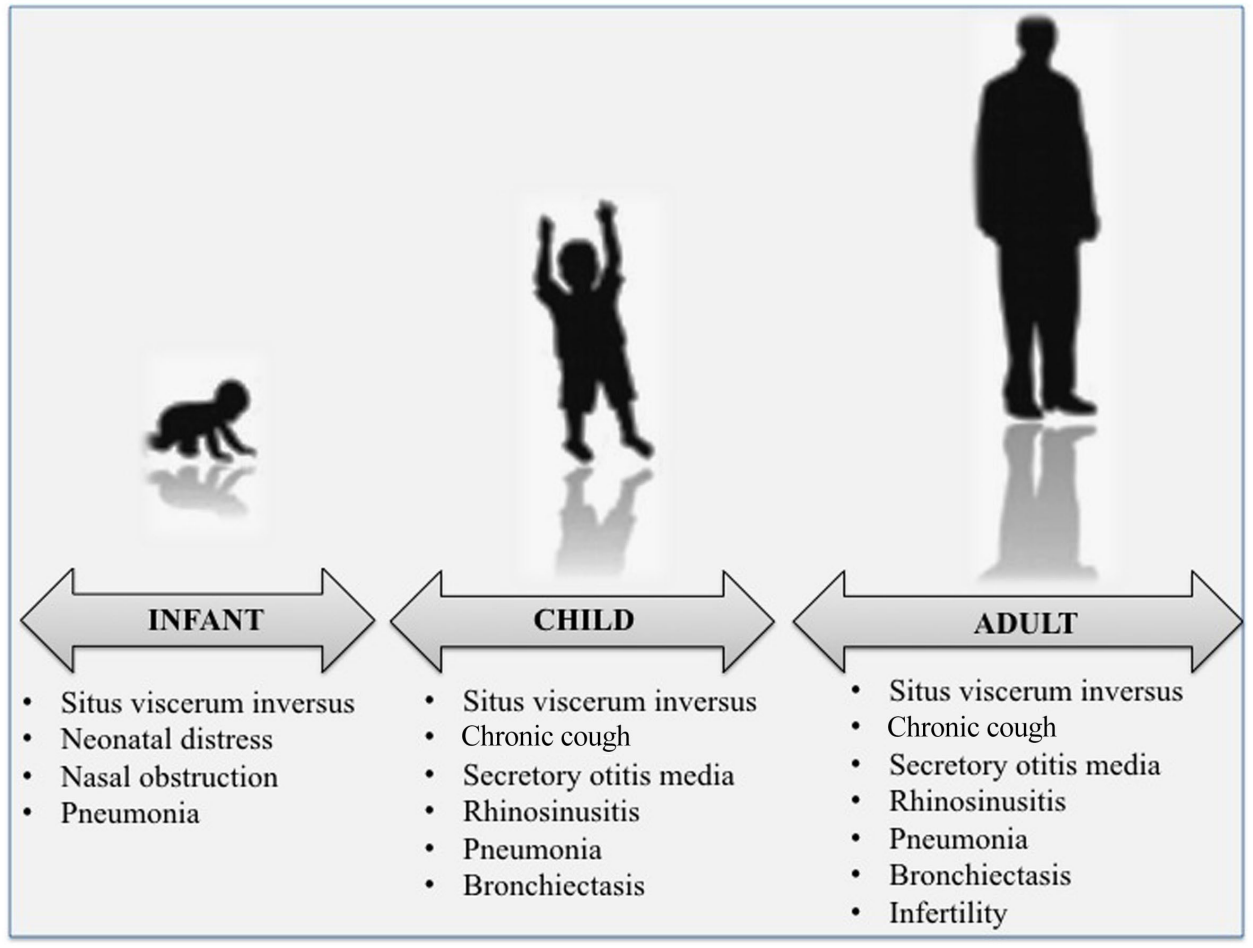
The classical clinical phenotypes of primary ciliary dyskinesia at various ages.

At all ages, the clinical phenotype of PCD is very wide (Figure 1). Respiratory manifestations are part of the classic description of the disease and are considered “*sine qua non*” features for the diagnosis. Main PCD manifestations include recurrent to chronic upper and lower respiratory tract infections that eventually complicate with bronchiectasis at older ages. Most symptoms occur on a chronic, daily basis and start soon after birth (1). Unfortunately, most of the symptoms or signs of PCD upper and lower airway disease are very common also in healthy children, and this is why the diagnosis is often made beyond infancy or childhood, with delayed start of follow-up and/or adequate treatment (19, 22).

Unexplained neonatal respiratory distress is a possible manifestation of PCD. Transient tachypnea of the newborn, starting soon after birth, is a well-known cause of respiratory distress in term and near-term newborns, and resolution by the fifth day of life is generally reported (23). Conversely, more than 75% of full-term neonates with PCD require continuous supplemental oxygen for days to weeks (24). The most stricking finding of a recent study was that pneumonia and multiple lobar collapse that require prolonged hospital stay may be very severe in newborns who will be later confirmed as PCD (25).

Persistent nasal obstruction is very common at all ages, as children and adults refer a daily year-round nose congestion evident yet in the neonatal period or in the first years of life (26, 27).

Chronic rhinorrhea complicated by anosmia, associated with recurrent secretory type otitis (glue ear), occur in 76–100% of PCD children (19, 28) and may lead to sleep-disordered breathing (29, 30). Chronic rhinosinusitis is frequently associated with hypoplastic frontal and sphenoid sinuses (31). Recurrent otitis media is a troublesome complaint in many PCD patients, with as much as 38% of the cases requiring more than 30 antibiotic courses in their life (20).

Lower airways are commonly involved in PCD.

**Figure 2:**
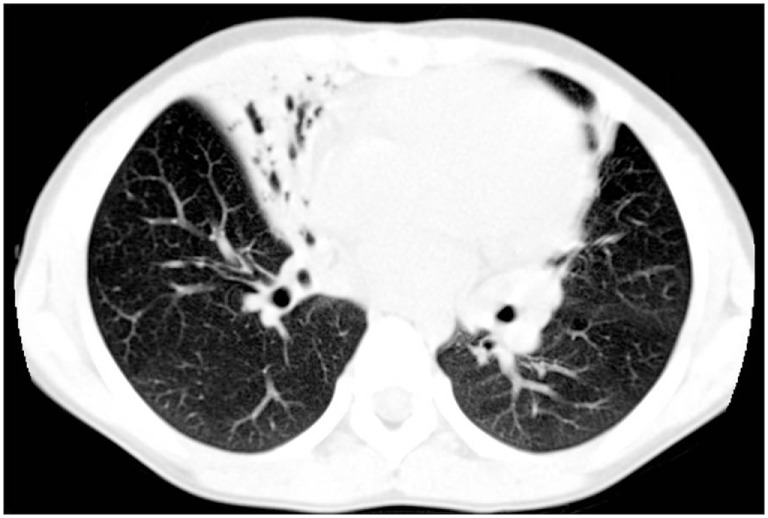
High-resolution computed tomography findings from a 7-year-old girl with primary ciliary dyskinesia. The scan demonstrates an area of consolidation both in the lingula and in the middle lobe, the latter also including bronchiectasis.

In preschool and school-age children daily wet cough due to repeated episodes of bronchitis and/or recurrent pneumonia is a universal finding (32), that may result in the development of chronic obstructive suppurative lung disease, with localized-to-diffuse bronchiectasis (1). The underlying cause of bronchiectasis was PCD in 1–17% of several pediatric case series (33–35). Although the development of bronchiectasis increases with aging (28), it has been reported even in toddlers with PCD (36). High-resolution computed tomography (HRCT) is a highly sensitive imaging modality for investigating PCD lung disease, and in particular to detect bronchiectasis (35, 37, 38) (Figure 2). However, HRCT involves larger radiation doses than the conventional X-ray procedure, and therefore its use in the follow-up of pediatric chronic lung disorders is controversial (39). Chest magnetic resonance imaging may be a valid alternative with a good-to-excellent agreement with HRCT findings (40, 41). In addition to wet cough and bronchiectasis, chronic asthma, generally unresponsive to maintenance treatment, is frequently reported at school-age and during adolescence (42). A mild to moderate obstructive pattern is a common finding at spirometry. Possible pathological changes mainly include bronchial obstruction, and altered lung mechanics secondary to repeated endobronchial infection (43).

Sputum cultures tipically yield oropharyngeal flora including *Haemophilus influenzae, Streptococcus pneumoniae*, and *Staphylococcus aureus* in the early childhood, while *Pseudomonas aeruginosa* (first smooth and then mucoid) and other Gram-negative pathogens such as *Klebsiella species* are prevalent in older PCD (44). Actually, mucoid *P. aeruginosa* may be found in 5% of PCD patients younger than 19 years (45). Although rare in childhood, non-tuberculous mycobacteria are reported in more than 10% of PCD adults (28).

As a consequence of abnormal sperm structure some, but not all, male patients with PCD have fertility problems (46). Sperm flagellum is a type of cilia. Therefore, abnormal ciliary structure may lead to the reduction or loss of the ability of the flagellum to swing, causing ultimately male infertility (7, 47). The most frequent ultrastructural defects of the sperm flagella are missing dynein arms, microtubular translocations, and lack of radial spokes (48). Having immotile sperm is common among affected males, and spontaneous pregnancy is rarely achieved unless through artificial insemination, including *in vitro* fertilization and intracytoplasmic sperm injection (49). For this reason, genetic counseling to couples pursuing assisted reproductive technology is mandatory, and genetic assessment of sperm is highly recommended prior to any clinical action. Females with PCD may experience an increased rate of ectopic pregnancy and decreased fertilization ability, because of impaired ciliary function in the oviduct (50, 51).

Situs inversus totalis is present in 50% of individuals with PCD (6). Heterotaxy, defined as an abnormality where the internal thoracoabdominal organs demonstrate abnormal arrangement across the left–right axis of the body is described in approximately 6% of the cases (52). Patients with heterotaxy may also have complex cardiac defects such as double outlet right ventricle, atrioventricular canal defects, atrial and ventricular septal defects, L-transposition of the great arteries, and tetralogy of Fallot (52, 53). The respiratory phenotypes of the PCD patients with heterotaxy are not different than those without heterotaxy (28). Other conditions as complex congenital heart disease, polycystic kidney and liver disease, hydrocephalus, biliary atresia, severe esophageal disease (esophageal atresia, severe reflux), and retinal degeneration, including retinitis pigmentosa, could also be associated in patients with PCD (54).

Data on growth of PCD patients are controversial. Chronic respiratory disease and long-term inflammation decrease IGF-I levels and compromise children’s growth, as demonstrated in cystic fibrosis (CF) (55, 56). At present, few studies that investigated growth in PCD using national and international reference values show conflicting results, with some suggesting impaired growth (57–59), and others reporting no differences (45, 60).

Despite it is well known that micronutrients and vitamins play a role in respiratory infections, data on their contribution in the inception or maintenance of PCD-associated airway infections are very scarce. Children and adults with stable PCD have deficient-to-insufficient serum vitamin D levels (61). Since, vitamin D has immunomodulatory properties and its deficiency may contribute to an increased risk of respiratory infections in PCD, studies aimed to evaluate the efficacy of vitamin D supplementation on the rate or severity of PCD infections exacerbations should be proposed at a multicenter level.

Compared to CF, the natural history of PCD lung disease is much less clear. Information on PCD disease progression is still incomplete, even though the mortality data are hard to interpret, as they are not age standardized. A recent retrospective study of 151 PCD adults with a median age of 35 years longitudinally followed for 7 years found an incidence of all-cause mortality of nearly 5%, and a respiratory mortality of 3.3% (62). Authors showed that older age at diagnosis was associated with impaired baseline FEV_1_ and increased *P. aeruginosa* colonization. Lung function decline, estimated at FEV_1_ decline of 0.49% pred per year, was positively associated with ciliary ultrastructure abnormalities, mainly microtubular defects (62).

The severity of lung disease in adults with PCD is highly variable, but is generally milder than in CF (14). However, a progressive course of PCD pulmonary disease is possible in mid-adulthood, with some patients developing an end-stage lung disease who may eventually require lung transplantation (28). Early studies have suggested relatively stable lung disease, in the absence of significant lung function decline (63, 64). Conversely, a recent study showed that only 57% of PCD patients followed at a single center over 5–30 years have a stable FEV_1_ and that lung function may progressively decline in approximately one-third of these (65). This finding has been recently confirmed by Werner et al. who documented progressive decline of FEV_1_ in 71 PCD cases from the international registry (21). Surprisingly, early referral to a PCD center may not be associated with better spirometry (60).

Traditionally, pulmonary function testing is the best non-invasive way of tracking the progression of the disease in chronic lung disorders, also including PCD. Spirometry results correlate with lung structure changes at HRCT, but the latter may progress despite little or no change in lung function (66). In recent years, there has been increasing focus on the lung clearance index (LCI), a measure of ventilation inhomogeneity that appears more sensitive than FEV_1_ in detecting early airway disease (67). Nevertheless, data in PCD on the relationship among LCI, spirometry, and lung structure changes at HRCT are conflicting (68, 69), and further investigation should be provided to clarify the role of LCI in the medium- to long-term progression of the disease.

## Diagnosis

A complete diagnostic work-up of PCD is mandatory if a positive family history of PCD is reported, and the latter can account up to 10% of all PCD diagnoses (19). Siblings of probands should have PCD excluded, particularly if they exhibit mild respiratory features that may not indicate PCD (1).

A 7-point questionnaire-based prediction tool (PICADAR) has been recently developed to predict the likelihood that a patient referred for evaluation of persistent wet cough has PCD (70). Authors proposed a final score that includes seven predictive variables, such as full-term gestational age, admittance to a neonatal unit, neonatal chest symptoms, persistent perennial rhinitis, chronic ear and hearing symptoms, situs abnormalities, and presence of a cardiac defect. Patients with a PICADAR score ≥10 have more than 90% probability of testing positive for PCD, while a score ≥5 indicates more than 11% chances of being diagnosed as PCD.

There is no single gold standard diagnostic test for PCD. Current diagnosis requires a combination of technically demanding investigations, including nasal nitric oxide (nNO), high-speed video microscopy analysis (HVMA), and transmission electron microscopy (TEM) (71).

Among the earliest diagnostic tests for PCD evaluation, the saccharine test and the investigation of mucociliary clearance by a radioactive tracer have been long used either inside or outside of specialized centers to demonstrate that mucociliary transport is impaired as a result of abnormal ciliary motion (1). However, the saccharin test may miss cases with dyskinetically beating cilia and the radioaerosol mucociliary clearance techniques are associated with radiation exposure albeit quite low. Therefore, the evidence appears too limited to recommend them (71).

Measurement of nNO is a helpful tool for screening PCD. Its levels are extremely low in PCD compared to healthy and disease controls (72). Possible explanation include a reduced biosynthesis of NO by paranasal sinuses or a possible increased consumption by superoxide anions or, alternatively, a sequestration in the upper respiratory tract within blocked paranasal sinuses or, finally, its biosynthesis or storage capacity is limited due to agenesis of the sinuses (73). nNO measurement should be used as part of the diagnostic work-up of schoolchildren over 6 years and of adults suspected of having PCD, preferably using a chemiluminescence analyzer and the velum closure technique, that achieves palate closure by exhaling through the mouth into a disposable resistor (74). This test is sensitive, rapid, non-invasive, and results are immediately available. Unfortunately, standardized methods to measure nNO are not appropriate for younger children, precisely the age group that would need urgent targeting for diagnostic measurement. In preschool children, nNO should be preferably measured using tidal breathing. Available data suggest that measurements of nNO correlate well with the values obtained at the plateau, but values are lower (71). The limitation is that breath-hold with velum maneuver can be difficult to obtain particularly by young children, and there is mounting evidence that simpler measurements, such as breath-hold without velum closure or sampling during tidal breathing, can discriminate between PCD and non-PCD also in younger children (75, 76). Diagnostic cutoff values for tidal techniques from preschool children are not currently available (77).

In older children, nNO analysis includes multiple methods of measurement and different cutoff values, making it difficult to provide definite thresholds in that age range. It has been reported that nNO cutoff value less than 77 nl/min strongly suggest PCD, with 98% sensitivity and 99% specificity (78). However, cases with demonstrated PCD may exhibit normal or even raised nNO levels (79). This indicates that patients with high clinical suspicion of PCD should be evaluated by additional diagnostic procedures other than nNO measurement (71).

Measurement of nNO can be obtained by stationary or handheld devices. Stationary devices are very commonly used, but are expensive and need frequent technical assistance (78). A handheld device simple to use and cheap has been developed, and a study found no difference between nNO obtained from stationary or handheld analyzer during silent and humming exhalation (80). A portable device equipped also for nNO analysis through the aspiration method is currently available (81, 82), but more experience is needed to validate its use.

**Figure 3:**
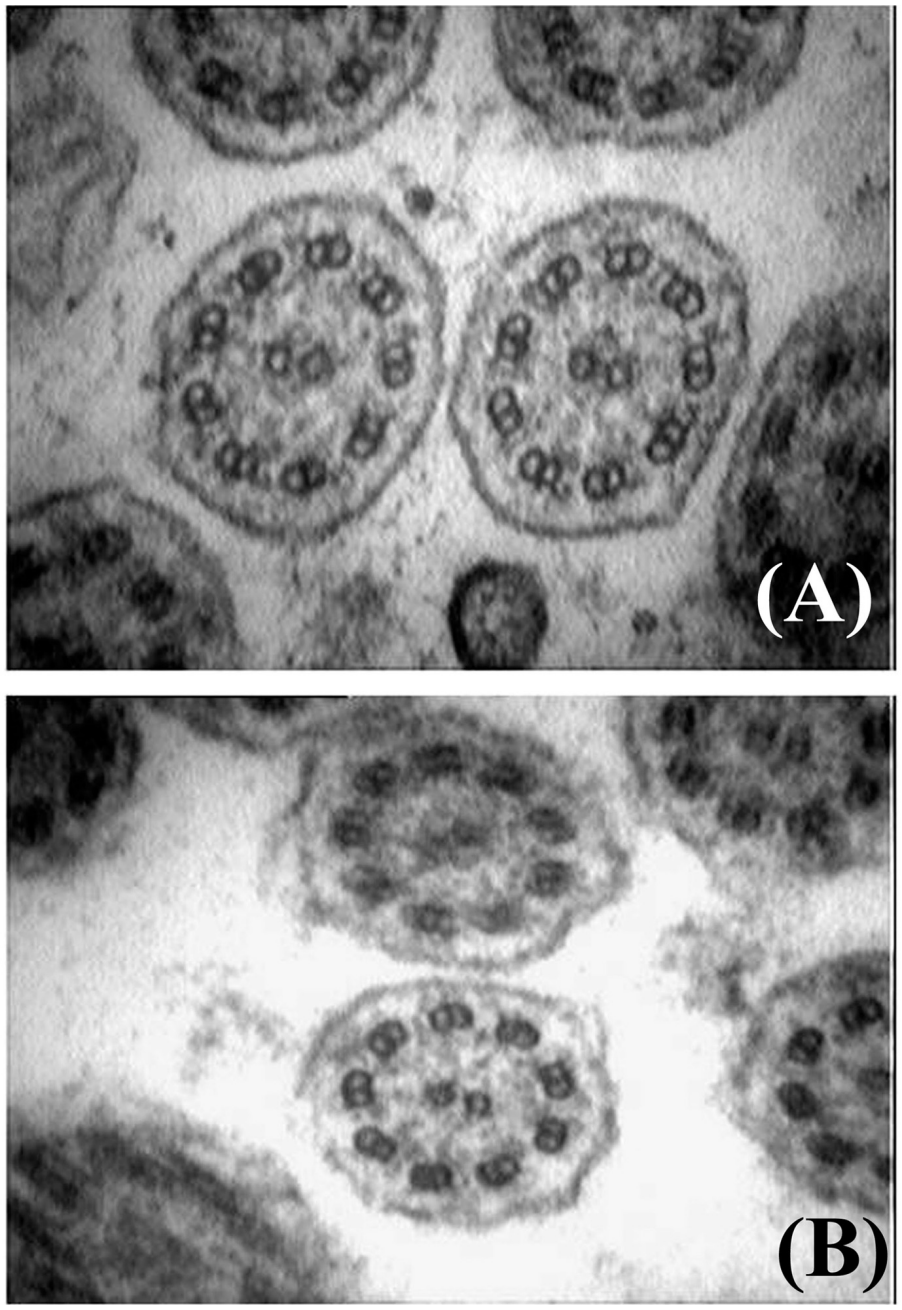
Electron microscopy findings showing normal cilia ultrastructure from an healthy subject **(A)**, and outer and inner dynein arms defect from a patient with primary ciliary dyskinesia **(B)** (courtesy of Dr. Mariarosaria Cervasio, Department of Advanced Biomedical Sciences, Anatomo-Pathology Unit, Federico II University, Naples, Italy).

Historically, a PCD diagnosis was based on analysis at TEM of ciliary cross sections from a nasal respiratory epithelium sample (83). This is usually obtained from the inferior turbinate of the nose by brush or curette biopsy or from the lower respiratory tract during bronchoscopy. Nasal brushing represents an elegant, simple, well-tolerated, and only minimally invasive way to collect the ciliated epithelium (84). The sample is chemically fixed with glutaraldehyde, processed, and cilia are analyzed using a transmission electron microscope (83, 85). Examination of the ciliary ultrastructure by electron microscopy remains a definitive diagnostic test for PCD (71). Nevertheless, TEM analysis can confirm but does not always exclude the diagnosis (86). It usually allows to identify PCD variants exhibiting a complete or partial absence of ODAs, combined ODA and IDA defects, and microtubular disorganization defects. Figure 3 shows cilia ultrastructure from a PCD patient, compared to the normal ultrastructure from a healthy subject. It has been reported that TEM fails to identify at least 30% of all PCD variants (57, 87), such as the nexin link (88, 89) or the central pair components defects (90), or those associated with DNAH11 mutations (80, 91, 92). Moreover, only a subset of radial spokes defects are identified by TEM (93), while false-positive diagnosis has been reported in cases with isolated IDA (94). Importantly, a number of ciliary abnormalities including absence of the central microtubular pair, disorientation of the cilia, and disarrangement of microtubules may be secondary to infection or inflammation as well (1). As secondary defects are absent after ciliogenesis in culture, this procedure has been recommended in order to distinguish between primay and secondary cilia defects (71). Finally, further diagnostic investigations should be performed in all cases with normal ultrastructure if the clinical history strongly suggests PCD (91).

Direct visualization of ciliary beat pattern (CBP) and frequency (CBF) by HVMA should be used as part of the diagnostic work-up of patients suspected of having PCD and in order to improve diagnostic accuracy of HVMA, CBF/CBP assessment should be repeated after air–liquid interface culture (71). CBF should not be used without assessment of CBP in diagnosing PCD. HVMA protocols differ among centers in many respects including sampling techniques, microscopes and cameras, temperature during analysis, software, and evaluation criteria. Videos are recorded using a digital high-speed video camera attached to an inverted phase-contrast microscope. Digital image sampling was performed at 120–150 frames per second (fps) and a 640 × 480 pixel resolution. A CBF of less than 11 beats per second (<11 Hz) has been suggested as a cutoff value, with only those with lower beat frequency proceeding to EM (95). On the other hand, HVMA is not sufficiently standardized to rule in or rule out PCD in isolation.

**Figure 4:**
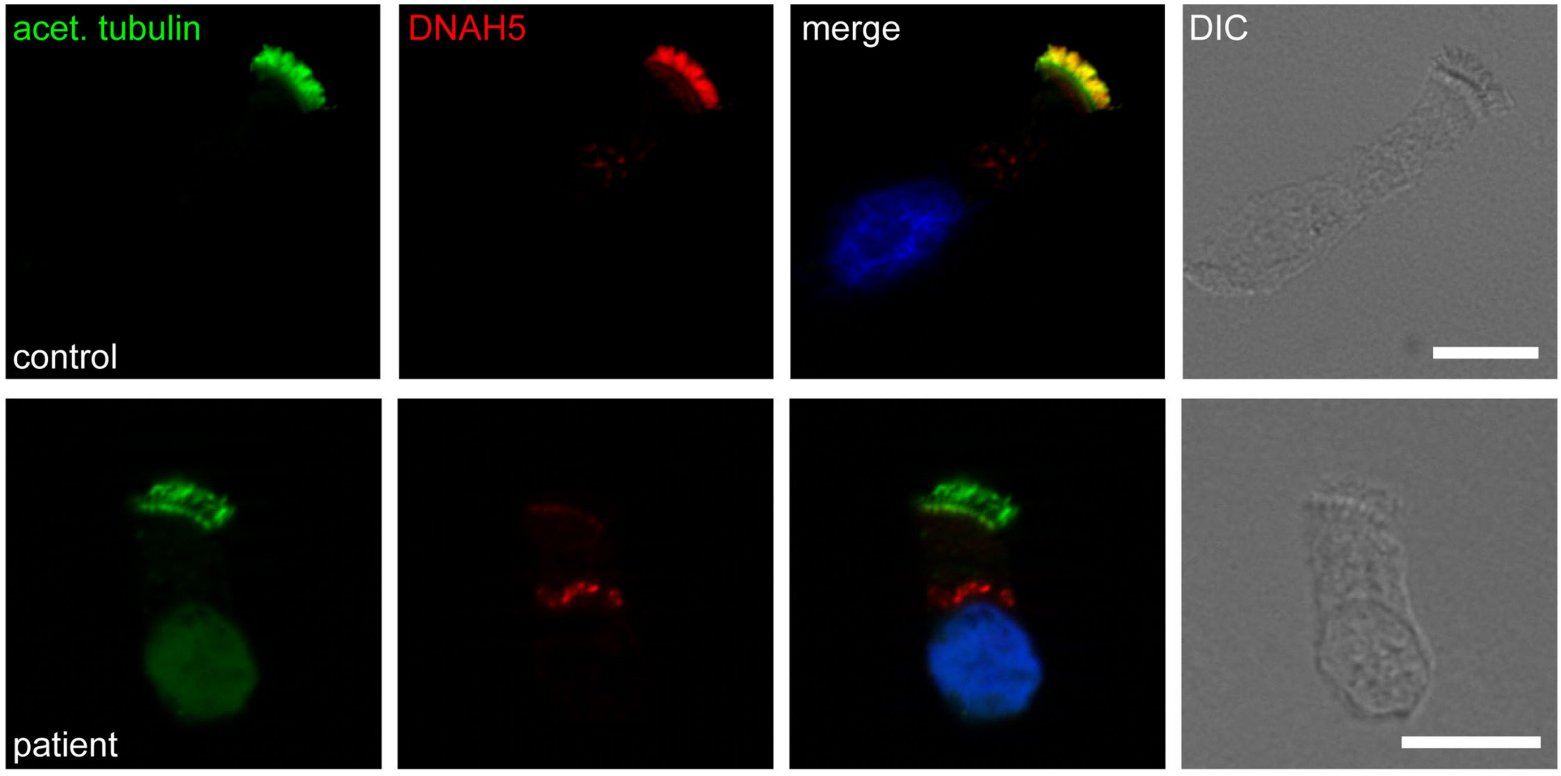
Immunofluorescence staining of human respiratory epithelial cells with DNAH5-specific antibodies (red) and antibodies against acetylated α-tubulin (green). Nuclei were stained with Hoechst 33342 (blue). Overlays and bright-field images are shown on the right. Whereas in healthy human respiratory epithelial cells (control; upper panel), both antibodies colocalize along the entire length of the ciliary axonemes, in an individual with an outer dynein arm defect (patient; lower panel), DNAH5 is absent.

High-resolution immunofluorescence (IF) analysis is an emerging tool to investigate the subcellular localization of ciliary proteins in respiratory epithelia (96) (Figure 4). It reliably identifies all ultrastructural abnormalities which are detectable by TEM (96–98), and additionally abnormalities of nexin links components (89) and radial spoke head proteins (10, 99–101). This technology has been adopted by an increasing number of laboratories and it is likely that further development will allow to recognize an increasing number of PCD variants.

## Genetics

Most PCD variants follow an autosomal recessive inheritance trait. The number of genes associated with PCD is still growing rapidly (Table 1).^1^ Some mutations leading to PCD are loss-of-function variants (102). Missense mutations can be found in a minority of cases. In these instances, it is often difficult to distinguish disease-causing mutations from rare polymorphisms. Most mutations are private. Clustering of mutations in specific genetic regions, as it is known from other genetic disorders, is less common. There is a good correlation between specific genetic mutations and their TEM, IF, and video microscopic phenotype (10). To date, only preliminary data have been published correlating genetic findings with distinct clinical phenotypes. Mutations in genes affecting central pair or radial spoke components (RSPH1, RSPH3, RSPH4A, RSPH9) as well as genes involved in the generation of multiple motile cilia (MCIDAS, CCNO) do not result in randomization of left/right body asymmetry. Therefore, affected individuals of those disease variants do not display situs abnormalities. This feature can be explained by the physiologic absence of the central pairs in the motile monocilia of the embryonic node. Patients with mutations in RSPH1 are reported to exhibit a milder clinical course (92); males with PCD due to CCDC114 mutations do not suffer from sperm immotility and therefore are not infertile (103). Subjects with reduced generation of multiple motile cilia are likely to have a more severe respiratory disease phenotype with lung failure at younger age (104, 105). Finally, a study has showed that lung disease is heterogeneous across all ultrastructural and genotype groups in 118 PCD patients from North America, but worse in those with biallelic mutations in CCDC39 or CDCC40 (45).

**Table 1:** Genes associated with primary ciliary dyskinesia and corresponding ultrastructure.

Gene^a^	Axonemal/cellular structure or function
*DNAH5, DNAI1, DNAI2, DNAL1, NME8 (TXNDC3)*	Outer dynein arm (ODA) subunit
*CCDC114, ARMC4, CCDC151, TTC25*	ODA targeting/docking factor
*DNAAF1 (LRRC50), DNAAF2 (KTU), DNAAF3, HEATR2, LRRC6, ZMYND10, DYX1C1 (DNAAF4), SPAG1, CCDC103, C21ORF59*	Cytoplasmic dynein arm assembly or transport factor
*RSPH1, RSPH3, RSPH4A, RSPH9*	RSPH subunit
*CCDC39, CCDC40*	NL/DRC factor
*CCDC164, CCDC65*	NL subunit
*DNAH11*	ODA subunit
*HYDIN*	CP subunit
*CCNO, MCIDAS*	*CCNO*: cytoplasmic centriole assembly and docking factor; MCIDAS: nuclear regulator of *CCNO* and *FOXJ1*
*OFD1, RPGR*	Functions related to non-motile cilia; role in motile cilia unknown

*^a^References can be obtained from authors*.

With the support of modern high-throughput genetic technologies, it is possible to identify disease-causing biallelic mutations in ~70% of affected individuals. However, given the complexity of diagnosing PCD using multiple different and repetitive tests, next-generation sequencing is a cost-efficient and effective diagnostic approach in many instances. It is very likely that further advances in PCD molecular genetics will continue to facilitate early diagnosis and treatment.

## Current and Future Treatment Strategies

Currently, therapeutic strategies of PCD are not based on validated disease-specific recommendations. Usually, patients are treated according expert opinion or to available evidence for CF, despite differences in the pathophysiology of the two disorders are evident.

The mainstay of treatment for PCD involves airway clearance, infection control and prevention, and the elimination of exposure to inflammatory triggers, also including passive smoke.

Different techniques guarantee airway clearance, including manual chest physiotherapy, postural drainage, autogenic drainage, active cycle breathing, and exercise (106).

Chest physiotherapists conduct deep breathing exercises, such as postural drainage combined with percussion and vibration and forced expirations, but the exclusive need for technical assistance may be time-consuming or uncomfortable (107). In addition to forced cough and breathing techniques, a variety of manual devices also exists that aid patients in improving mucus clearance. These include positive expiratory pressure (PEP) valves, and mouthpiece or chest wall oscillating devices (108). PEP devices, which give a constant back pressure to the airways during expiration, provide a pressure behind the mucus that push it out of the lungs and is widely used also in CF patients (109). There is no clear evidence that PEP is a more or less effective intervention than other forms of physiotherapy (110). High-frequency chest wall oscillation involves an inflatable vest that is attached to a machine, generating extrathoracic oscillations at variable frequencies and intensities, which are transmitted to the airways, promoting coughs or huffs (107). In conclusion, irrespective of the chosen modality and despite the lack of evidence-based comparisons of the various techniques, routine daily physiotherapy is strongly recommended in PCD (71).

Physical exercise should be prescribed to all subjects with obstructive pulmonary disease for improving respiratory muscle strength and maintaining lung health. It has been reported that a high proportion of PCD cases (79%) have limitations in performing vigorous activities, and approximately 50% spend less than 3 h per week doing physical activity, thus suggesting that PCDs are quite inactive (61). Performing exercise prior to airway clearance may more significantly enhance mucociliary clearance and is more effective as bronchodilator stimulus than β_2_-agonists drugs (111). Actually, compared to healthy individuals, patients with PCD have also significantly lower peak oxygen uptake measured by cicloergometry (112, 113).

Nebulized inhalation is a common procedure to help moisten and dilute viscous airway secretions, and thereby facilitates mucoclearance techniques (114). Inhaled hypertonic saline is used in the treatment of bronchiectasis for enhancing mucociliary clearance (115). In a randomized controlled trial of 7% hypertonic saline versus isotonic saline, adults with non-CF bronchiectasis reported increased ease of expectoration, reduction in antibiotics use and emergency health care visits over a 3-month period (116). Conversely, no significant change of spirometry, as well of sputum colonization or quality of life, was reported (117). A recent randomized controlled study of a small sample of adult PCD patients treated with inhaled hypertonic saline for 12 weeks neither improved quality of life nor significantly affected spirometry or airway inflammation (118). Further, larger studies also including children are needed to confirm these results.

During infections, DNA and actin released by neutrophils accumulation increase sputum viscosity in the airways. Recombinant human DNase I (rhDNase) cleaves extracellular DNA, decreasing the DNA concentration, and thereby decreasing sputum viscosity (119–121). Inhaled rhDNase improves FEV_1_ percent predicted in CF patients, and CF physicians often recommend it in their clinical practice (122). Neutrophilic airway inflammation has been reported in PCD (123). Until now, few PCD studies have showed significant clinical benefits of a trial with inhaled DNase (124–126). At present, rhDNase is not recommended in PCD, and larger studies are needed to confirm its efficacy in PCD. Uridine-59-triphosphate (UTP) may enhance clearance during cough stimulating chloride^−^ secretion and mucin releasing by goblet cells. Several years ago, a small study demonstrated that aerosolized UTP improves whole lung clearance measured by gamma scintigraphy during forced cough in 12 adolescents and adults with PCD, without any adverse effects (127). Unfortunately, no further studies were published on this issue. Mannitol also affects mucociliary clearance and is often prescribed to CF patients because it creates an osmotic drive for water to move into the airway and hydrate secretions (128). Data that sustain inhaled mannitol in PCD are lacking. Mannitol 400 mg inhaled twice daily for 12 months in adults with clinically significant non-CF bronchiectasis did not reduce exacerbation rates, but quality of life significantly improved (129). These findings indicate that a randomized clinical trial of inhaled mannitol might be proposed to PCD patients as well.

All patients with PCD should have routine clinical visits for spirometry monitoring and respiratory culture surveillance through sputum or oropharyngeal cultures (130). At any age, a minimum of two to four visits per year are recommended (131), and in case of respiratory exacerbations, antibiotics selected should be prescribed accordingly to culture history and microbial sensitivity.

Studies of CF and non-CF bronchiectasis also including some patients with PCD have demonstrated that systemic antibiotics are effective at treating “exacerbations” of lung disease (132, 133). Either respiratory tract symptoms including changes in cough, sputum production, respiratory rate, and work of breathing, or a decline in FEV_1_% predicted may be considered as reliable markers of a respiratory exacerbation in PCD. While mild exacerbations may be treated with oral antibiotics and increased aggressive airway clearance, severe or refractory exacerbations may require intravenous antibiotics and inpatient hospitalization. A duration of 14–21 days of antibiotic therapy is recommended in PCD, according to what is reported in CF and non-CF bronchiectasis (134–136). The selection of antibiotics should be made on the basis of the most recent sputum culture results and would take into account the airways colonization history of the individual patient. Macrolides are a class of antibiotics that deserves particular attention by pulmonologists. Macrolides play antibacterial activity at concentrations lower than those required to kill the infecting or colonizing bacteria (137). In addition to this, macrolides anti-inflammatory and immunomodulatory properties are also well recognized (138). Three randomized, double-blind, placebo-controlled studies of non-CF bronchiectasis, also including few cases with PCD demonstrated that azithromycin or erythromycin taken for 6–12 months led to significant decrease in exacerbation rate and reduced the decline in lung function (139–141). A PCD multicenter, double-blind, randomized, placebo-controlled trial is currently evaluating the efficacy of oral azithromycin administered three times a week for 6 months on the frequency of respiratory infectious exacerbations (142). Results will hopefully clarify whether macrolides may play a role also in PCD.

Cycled or regular inhaled or oral antibiotics may be a treatment option in patients with moderate to severe lung disease that fail eradication strategies and continue to be symptomatic. Despite there are no published studies, inhaled antibiotics are also an option for PCD respiratory exacerbations, but these are usually reserved for patients with *P. aeruginosa* infection. The use of inhaled tobramycin (300 mg nebulized twice daily) for a 28-day period should be considered upon the first evidence of *P. aeruginosa* growth (143).

Pulmonary surgical resection (i.e., segmentectomy or lobectomy) may be considered with caution in the presence of diffuse lung disease and can be considered only when a disproportionately burdened region of the lung has failed medical management of bronchiectasis, and there is a significant decline in patient’s health due for instance to severe hemoptysis.

If end-stage lung disease develops, lung transplantation may be an option in PCD. Particular attention must be payed in lung transplant evaluation of patients with PCD as situs abnormalities may pose a barrier in donor lung selection and require advanced surgical planning (144, 145).

The management of PCD ear and nose disease does not differ from that of the lung disease. Close follow-up also of ear–nose–throat district may help undoubtedly to avoid local or systemic complications. Recurrent or persistent otitis media with effusion may lead to chronic otitis media and hearing loss, and frequent use of antibiotics or even middle ear surgery may be ultimately decided (146). Whether or not tympanostomy tube placement may improve hearing loss is controversial, as it significantly increased the risk of chronic otorrhea and infection (147). Patients with sinus disease refractory to medical management may benefit from endoscopic sinus surgery (148).

It is also critical to avoid exposure to inflammatory triggers such as tobacco smoke, and therefore, patients and their family members should receive smoking cessation counseling.

Infection prevention is strongly recommended in PCD, as in all chronic respiratory diseases. Children and adults with PCD have increased risk for pneumococcal disease (148), and therefore, CV13 vaccinations are recommended followed by PPSV23 vaccination. Influenza vaccines are also recommended on an annual basis (149), and additional vaccinations are recommended as per the routine schedules of patients’ geographic regions of treatment.

As far as the future, an improved understanding of the underlying genetics and phenotyping of PCD will also hopefully lead to novel therapeutic strategies. A great expectation has originated from the recent study by Pifferi et al. who first applied the “gene editing” to PCD; thus, they restored DNAH11 gene function *ex vivo* by replacing the inactivating mutation with wild-type sequence in the diseased cell (150). A new exciting era is cheerfully rising from genetic studies that will result in improving the outcome of affected patients.

The optimal integration of multiple skills in an enlarged team, which hopefully includes pediatricians, pulmonologists, chest physiotherapists, geneticists, biologists, cardiologists, radiologists, andrologists, and ENT surgeons, is essential to provide the most appropriate care to children and adults with PCD. All these specialists do have the unique opportunity to play an integrated role in the multidisciplinary approach to the disease.

## Psychological Issues

Physicians who take care of children with PCD should take into valuable account the psychosocial impact of the disease. PCD leads to chronic respiratory symptoms and progressive loss of lung function, and this has a great impact on patients’ health and on style and quality of life of their families (151). As a chronic disorder, PCD is a stressful condition particular during adolescence and young adulthood because of the psychological effects of the chronic burden on the intrafamiliar relationships. Fortunately, patients who are diagnosed early, and hence receive more treatment for their condition, have better clinical outcome (152).

## Ethics Statement

The authors declare that written informed consent was obtained by the patients or healthy controls or their legal guardians for images to be published.

## Author Contributions

VM has made substantial contributions to conception and design, has been involved in drafting the manuscript, and has given final approval of the version to be published. CW has been involved in drafting the manuscript and has given final approval of the version to be published. FS has made substantial contributions to conception and design, has been involved in drafting the manuscript and revising it critically for important intellectual content, and has given final approval of the version to be published.

## Conflict of Interest Statement

The research was conducted in the absence of any commercial or financial relationships that could be construed as a potential conflict of interest.

## Footnotes

^1^Available from: https://www.omim.org/entry/244400 (2017).
